# Violence in first-episode psychosis: evidence from an early intervention in psychosis programme

**DOI:** 10.1192/bjo.2023.564

**Published:** 2023-09-19

**Authors:** Oihane Mentxaka, María Recio-Barbero, Eunate Arana-Arri, Rafael Segarra

**Affiliations:** Department of Psychiatry, Cruces University Hospital, Barakaldo, Spain; Early Stages of Psychosis Group, Biocruces Bizkaia Health Research Institute, Barakaldo, Spain; and Department of Neurosciences, University of the Basque Country UPV/EHU, Leioa, Spain; Early Stages of Psychosis Group, Biocruces Bizkaia Health Research Institute, Barakaldo, Spain; and Department of Neurosciences, University of the Basque Country UPV/EHU, Leioa, Spain; Biocruces Bizkaia Health Research Institute, Barakaldo, Spain; Department of Psychiatry, Cruces University Hospital, Barakaldo, Spain; Early Stages of Psychosis Group, Biocruces Bizkaia Health Research Institute, Barakaldo, Spain; Department of Neurosciences, University of the Basque Country UPV/EHU, Leioa, Spain; and Centro de Investigación Biomédica en Red de Salud Mental CIBERSAM, Institute of Health Carlos III, Madrid, Spain

**Keywords:** Psychosis, violence, offender, victimisation, criminality

## Abstract

**Background:**

Psychotic disorders are frequently associated with a public perception of dangerousness and belligerence. This situation has contributed to the social stigmatisation of people with severe mental illness and the resulting discrimination that this scenario entails. Despite efforts to demystify such disorders, the association between violent behaviour and psychosis remains unclear.

**Aims:**

To explore the incidence of the main types of violent offences in a cohort of patients presenting with first-episode psychosis (FEP).

**Method:**

Participants were recruited from the First Episode Psychosis Intervention Program (CRUPEP) cohort between 2009 and 2016. The main clinical variables were collected, including medical-forensic records of participants registered at the Basque Institute of Forensic Medicine (BIFM), to identify any violent acts in which participants were involved, either as victims or as offenders.

**Results:**

Overall, 79.5% (*n* = 182) of the participants had no record of violent crime or offence recorded in the BIFM. Annual crime rates for the 2009–2016 period show a decreasing trend in both the general population (IRR = 0.981, 95% CI 0.978–0.983, *P* < 0.001) and in the FEP group (IRR = 0.019, 95% CI 0.012–0.028, *P* < 0.001); this pattern is more pronounced in the FEP group. Victimisation accounted for the vast majority of reported incidents; nevertheless, participants who had committed violent offences were mostly involved in intrafamily violence.

**Conclusions:**

Individuals with FEP were not involved in a higher number of crimes than the general population. The types of violent acts committed by people with FEP were heterogeneous, with extreme violence being particularly uncommon.

Public perception of severe mental illness is often associated with dangerousness and a proclivity to be involved in violent and criminal behaviours.^1,2^ Although efforts have been made to combat this stigma, people with mental disorders are often perceived as impulsive and aggressive,^3,4^ or even socially inappropriate.^5^ In fact, increased public and media attention linking violent and criminal behaviour to mental disorders has fostered such perceptions.^6,7^ Thus, it is common that some of the most notorious violent events reported in mainstream media are explicitly linked to psychiatric disorders in general^8^ and to schizophrenia in particular.^9,10^ Such a situation has fostered the societal stigma of dangerousness for people with severe mental illness, with consequent discrimination.^5,11^ Despite efforts to demystify such disorders, the link between violent behaviour and mental disorders remains unclear. In this regard, several reports that have investigated this issue have not yielded conclusive results.^12–14^

Several studies have reported a positive relationship between schizophrenia and violence.^9,15^ In this respect, people with schizophrenia seem more prone to be involved in at least one crime in their lifetime than the general population,^16^ and this rate is further increased in those presenting with comorbid substance misuse.^9,16^ However, such connections are not necessarily causal, but instead suggest a possible link between having schizophrenia and a tendency to be involved in violent offences.^17^ In this regard, despite comprehensive reviews in the field having pointed out that people with psychosis have an increased risk of committing violent offences, the actual proportion of societal violence attributable to this group is small**.**^16^ Furthermore, when considering people with first-episode psychosis (FEP), the evidence shows inconclusive results, suggesting that a moderate percentage commit violent acts of a non-severe nature,^18,19^ particularly those who have not been previously treated with antipsychotics.^20^ Despite the widespread link of psychosis with violence, there is growing evidence that people with FEP are also victims of violence.^21^ In this research study, we aim to fill this knowledge gap by exploring the incidence of violent offences in a cohort of individuals experiencing FEP, considering the main types of offences documented and whether they have been victims or perpetrators of violent offences.

## Method

### Participants

Participants were recruited from the First Episode Psychosis Intervention Program (CRUPEP) clinical cohort, a specialised early intervention in psychosis programme at the Cruces University Hospital (Barakaldo, Spain). The programme covers an area of 150 000 inhabitants comprising urban districts of lower-middle socioeconomic status. All individuals consecutively referred by presenting with a first episode of affective or non-affective psychosis according to the DSM-IV between 2009 and 2016 and followed up during the first 5 years after presentation were assessed for inclusion in this study. Inclusion criteria were: (a) age over 18 years, (b) follow-up during the study period and (c) written informed consent. The exclusion criteria were (a) no previous antipsychotic treatment or, if prescribed, less than 1 month prior to referral to CRUPEP, (b) presence of organic brain disease, and (c) IQ <70.

The authors assert that all procedures contributing to this work comply with the ethical standards of the relevant national and institutional committees on human experimentation and with the Helsinki Declaration of 1975, as revised in 2008. All procedures involving human participants were approved by the Integrated Healthcare Organisation (OSI) Ezkerraldea-Enkarterri-Cruces Ethics Committee (study ethical approval number CEI-E 17/06).

### Measures

The main clinical variables, such as sociodemographic data, including the MEDEA socioeconomic deprivation index,^22^ psychiatric diagnosis based on DSM-IV and other relevant clinical information, were collected from paper and electronic medical records. In addition, the medical-forensic records of patients registered at the Basque Institute of Forensic Medicine (BIFM) were retrospectively reviewed, to retrieve any relevant information regarding offences (violent acts) in which the study participants were involved, either as victims or offenders. This review covered any record registered at the BIFM before the onset of psychosis up to inclusion in the study. Data were also collected concerning traffic accidents that required the intervention of the Medical-Forensic Clinic in cases in which evidence of criminality was found. Typification of main offences according to the Spanish Penal Code is presented in Supplementary Appendix A, available at https://doi.org/10.1192/bjo.2023.564.

### Classification of participants according to the BIFM records

Participants were classified into three subgroups depending on whether they had been only a victim of violent offences (‘victim only’), only a perpetrator of violent offences (‘offender only’) or both a victim and a perpetrator (‘victim-offender’).

### Statistical analysis

The main categorical variables were described using frequencies and percentages, whereas continuous variables were described using means and standard deviations. Statistical comparisons among categorical variables were performed using the chi-squared test. For quantitative variables, either Student's *t*-test or the Mann–Whitney *U*-test was used. A Poisson linear regression model was used to estimate the rates of criminality and the incidence rate ratio (IRR). A confidence interval of 95% was used, with 0.05 as a threshold for statistical significance. Data were analysed using IBM SPSS Statistics for Windows, version 21.0.

#### Estimation of crime rates

Crime rates were calculated as the number of known penal infractions over the total population. For the purpose of this study, annual crime rates of the entire OSI Ezkerraldea-Enkarterri Cruces population during the period 2009–2016 were calculated as well as crime rates of our FEP cohort. These values were compared to determine whether the FEP cohort committed more penal infractions than the average population (for further information see Supplementary Appendix B):
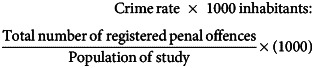


Annual crime rates for the FEP cohort were calculated as the total number of offences reported each year of the study divided by the total active patients of the cohort:
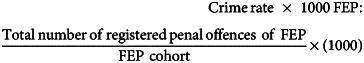


## Results

The FEP cohort comprised 229 individuals who consented to participate in the study. The main characteristics of the subgroup (*n* = 49) with a record of violence at the BIFM are detailed in Table 1. Patient electronic records documented that 79.5% (*n* = 182) of the group registered no record of violent offences according to the BIFM.

**Table 1:** Sociodemographic and clinical variables according to the three subgroups of participants with first-episode psychosis and a record of violence

	‘Victim-offender’ (*n* = 5)	‘Offender only’ (*n* = 14)	‘Victim only’ (*n* = 30)	*P*
Gender, male: *n* (%)	4 (80)	14 (100)	21 (70)	0.302
Age when violence recorded, years: mean (s.d.)	27.60 (13.9)	28.23 (8.85)	26.70 (11.5)	0.558
Education level, *n* (%)				
Not completed elementary school	4 (80)	6 (42.9)	8 (26.7)	
Elementary school	1 (20)	5 (35.7)	11 (36.7)	0.182
Secondary education	0	3 (21.4)	11 (36.7)	
Unemployed (yes), *n* (%)	4 (80)	10 (71.4)	12 (40)	0.235
Clinical diagnosis, *n* (%)				
Schizophrenia	3 (60)	7 (50)	15 (50)	
Bipolar disorder	1 (20)	4 (28.6)	6 (20)	
Schizoaffective disorder	0	2 (14.3)	1 (3.3)	0.706
Delusional disorder	0	0	3 (10)	
Other psychotic disorders	1 (20)	1 (7.1)	4 (13.3)	
Age at onset of psychosis, years: mean (s.d.)	27.60 (12.5)	25.75 (9.87)	29.90 (11.15)	0.406
Violence records registered at BIFM, mean (s.d.)	2.60 (0.89)	1.71 (1.20)	1.23 (0.68)	0.003
Violence records registered at BIFM, *n* (%)				
1	–	8 (57.1)	25 (83.3)	
2	1 (20)	3 (21.4)	2 (6.7)	0.001
>3	4 (80)	3 (21.4)	3 (10)	
Prior victimisation (yes), *n* (%)	2 (33.3)	2 (15.4)	2 (7.4)	0.329

BIFM, Basque Institute of Forensic Medicine.

The annual crime rates during the period 2009–2016 showed a decreasing trend both in the general population (IRR = 0.981, 95% CI 0.978–0.983, *P* < 0.001) and in the FEP cohort (IRR = 0.019, 95% CI 0.012–0.028, *P* < 0.001); this tendency was more pronounced in the latter case. Particularly, no criminal offence was committed in 2012 by the FEP cohort according to the Basque Country Department of Security. In addition, the variability in crime rates was greater in the FEP group than in the general population.

**Fig. 1:**
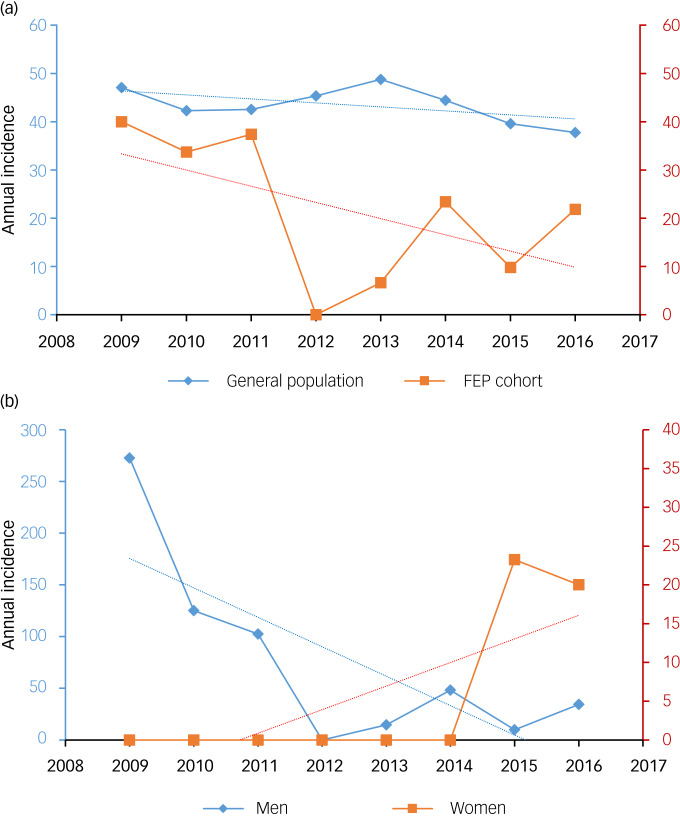
Annual crime rates (a) in the general population and first-episode psychosis (FEP) cohort and (b) in the FEP cohort, disaggregated by gender.

In Fig. 1, we present the evolution of crime rates for the FEP cohort during the years of the study. From 2009 to 2016, crime rates showed a downward trend in males in the FEP cohort (IRR = 0.401, 95% CI 0.025–0.062, *P* < 0.001). For females, it is noteworthy that during the first 5 years of the study, no criminal behaviour was recorded (IRR = 0.101, 95% CI 0.025–0.0405, *P* < 0.001), with an upward trend thereafter.

### Participants with a record registered at the BIFM

We subdivided the group of participants who had records of violence registered at the BIFM by considering whether they had only been a victim (‘victim only’), only been a perpetrator (‘offender only’) or had shifted from victim to perpetrator (‘victim-offender’). Table 1 shows the main clinical variables collected from the forensic reports for each subgroup. Of the 49 participants who had records of violence registered at the BIFM, either as a victim or as an offender, over half (59.6%) had never been a perpetrator, compared with 29.2% who had never been victims. In this regard, our sample had at some point in their clinical follow-up been offenders, with a mean of 0.9 (s.d. = 1.04), compared with an overall mean of 0.90 (s.d. = 0.78) for being victims.

Considering main sociodemographic characteristics, it is worth noting that 80% of the ‘victim-offenders’ subpopulation had not completed elementary education. Likewise, nearly half of the ‘offender only’ subgroup had not completed elementary school (42.9%). Here, it should be highlighted that none of the participants who had records of violence had completed post-secondary education. With regard to employment status, Pearson's chi-squared test indicated significant differences among the three groups (*P* = 0.011). Concerning the MEDEA socioeconomic deprivation index, 100% of the victim-offender subpopulation lived in districts with a MEDEA quintile index of 4 or 5, which is associated with a greater socioeconomic inequality and poverty (results not shown).

Over half of the participants in each of the three groups were diagnosed with schizophrenia, which was the most common diagnosis. In addition, 89.3% (*n* = 42) of those who had one or more violence records did not report victimisation in childhood. Childhood victimisation was defined as having experienced physical violence or intrafamily violence before the age of 16. Interestingly, when performing a gender subanalysis, males represent 100% of ‘offenders’, 80% of ‘victim-offenders’ and 70% of ‘victims only’ (*P* = 0.11).

### Main violent offences registered at the BIFM

After analysing all records obtained from participants who had a violence record registered at the BIFM, we found that the average of violence records was 1.23 (s.d. = 0.68). As presented in Table 1, among the participants who were ‘offenders only’, 57.1% had at least one record and 21.4% had two records, which gives an average of 1.71 records per patient (s.d. = 1.20). Notably, of the participants who shifted from ‘victim to offender’, 20% have two records and 80% have three or more records. Hence, individuals who moved from ‘victim to offender’ had an average of 2.6 records (s.d. = 0.89). Finally, 83.3% of the participants identified as ‘victims only’ had a single record and 6.7% had two records. These differences were statistically significant (*P* = 0.001).

When considering the number of violent acts committed by participants and reported in the BIFM (Table 2), more than half (66.7%, *n* = 8) involved violent acts classified as intrafamily violence, followed by burglary with force or robbery with violence or intimidation (16.6%, *n* = 2). In the case of participants who were victims of violent offences, injuries were the most frequent cause in more than half of cases (58.6%, *n* = 17), followed by intrafamily violence (24.1%, *n* = 7). It is worth mentioning that more than two-thirds of the ‘victim' subgroup suffered aggressions from people outside the family (70%, *n* = 21). Conversely, the ‘aggressor’ subgroup commits aggression within the immediate family circle on almost 60% of the occasions (*n* = 8).

**Table 2:** Type of penal infractions committed by participants with first-episode psychosis according to Spanish penal code

Type of offence	*n* (%)
As offender	
Intentional homicide	1 (8.3)
Intrafamily violence	2 (16.7)
Recurrent intrafamily violence	6 (50)
Burglary with force	1 (8.3)
Robbery with violence or intimidation	1 (8.3)
Assaults on the authority	1 (8.3)
Total	12 (100)
As victim	
Attempted homicide	1 (3.4)
Intrafamily violence	3 (10.3)
Recurrent intrafamily violence	4 (13.8)
Injuries	17 (58.6)
Robbery with violence or intimidation	1 (3.4)
Theft or misappropriation of motor vehicles	1 (3.4)
Offences against public health	2 (6.9)
Total	29 (100)

## Discussion

This study aimed to assess whether people with FEP are more likely to commit violent crimes than the general population. To do so, we calculated the annual crime rates registered for a selected cohort during the years 2009–2016 and compared them with the annual crime rates for the general population in a selected area during the same period.

### Crime rates

Our results reflect a greater variability in the crime rates of our cohort of people with FEP compared with the population of the local health services catchment area (OSI Ezkerraldea-Enkarterri-Cruces), as well as the general population. Therefore, we stress the need to further investigate the presence of additional factors that might affect the shift to offending in people with FEP. Based on the results of this study, we conclude that people with FEP have comparable or even slightly lower crime rates than the general population. These findings lead us to suggest that factors associated with violence in early psychotic episodes may be comparable to those found in the general population.

Indeed, we found that during the study period, people with FEP followed up in CRUPEP had a crime rate varying between 0 and 30.56 per 1000 inhabitants. In contrast, crime rates in the OSI Ezkerraldea-Enkarterri-Cruces catchment area showed a variable range between 37.76 and 48.76 per 1000 inhabitants. Therefore, and despite the small sample of people with FEP presenting violent records, crime rates show a greater downward trend in people with FEP in comparison with the trend found in the general population.

Such results might be partly explained by the fact that CRUPEP, over the years, has progressively enrolled and provided adequate treatment at earlier stages for the vast majority of people with FEP in our demographic catchment area. Based on the annual crime rates published by the Spanish Ministry of the Interior, a downward trend was observed, from 50 per 1000 inhabitants in 2009 to 43.2 per 1000 inhabitants in 2016.^23^ Over the years, crime rates have remained lower in people with a FEP compared with their representative population, which in this case considers the inhabitants in the demographic area included in our area of attention.

In particular, no patients followed up in our study committed a violent criminal offence in 2012, compared with the general population of the OSI Ezkerraldea, where criminal offences are registered annually. We consider that this result may be influenced by the small size of our sample of people with FEP, whose tendency to commit criminal offences has been analysed on a year-by-year basis.

### Demographics

Regarding socioeconomic status, it is noteworthy that approximately two-thirds of the sample resides in districts with a MEDEA 4–5 quintile (those with the highest societal inequality), which further supports the findings of previous works linking low socioeconomic status and poverty to psychosis and violence.^24,25^ These results are of special relevance considering that the average MEDEA index in the Basque Country is in quintile 3, indicating that, on average, the population is in a middle position in terms of socioeconomic inequalities in small areas.

### Types of violent offence

No significant differences were found in the types of violent offence committed by our sample of people with FEP. However, the most frequent type could be classified as intrafamily violence, which includes abuse in the family. This finding is in line with previous reports in the literature showing that violence is mainly directed towards family members and takes place mostly at home.^26^

Concerning the risk of people with FEP committing acts of extreme violence such as homicide, we found a single case of intentional homicide and this corresponded to the first violent offence committed by an individual with FEP before the start of the psychiatric follow-up. Furthermore, when a person with FEP is the victim, we found that in a 75% of the events, individuals suffered aggression defined in the Spanish Penal Code as ‘injuries’ and ‘crimes of torture and against integrity’, where, once again, intrafamily abuse is predominant and in the vast majority of cases, individuals are not following psychiatric treatment at the time of the aggression.

This leads us to re-emphasise that early detection of a first psychotic episode through specialised units such as CRUPEP, as well as the implementation of strategies that ensure adequate adherence to treatment such as antipsychotics in depot formulations, or even involuntary out-patient treatment in selected cases, play a primary role in the prevention of violence in people with psychosis.

We conclude that the types of crime committed by the FEP cohort differed from those committed by the general population. Extreme violence remains a minority, with the vast majority committing acts of mild violence that mostly take place in the family environment. As an explanation for this finding, we underline the progressive de-institutionalisation of the psychiatric system, along with the current shift towards community psychiatry. As a result, psychiatric patients not only reside with their relatives but the family is also the primary source of external support, which may result in an overburden on family members and a deterioration of family dynamics.

### Limitations and strengths

The present study has several limitations, which, nevertheless, may represent future lines of research. First, despite the longitudinal nature of the data, covering a time period from 2009 to 2016, the sample of participants who had records of violence (as victims, perpetrators or both) was small. Second, it should be borne in mind that the CRUPEP programme provides care for a high percentage of people who experience a first psychotic episode in our healthcare area. However, for a variety of reasons beyond the scope of this work, it is possible that a certain number of individuals do not enter CRUPEP, owing to private healthcare, or may never attend any mental healthcare service.

Another limitation of this study is its retrospective nature, given that the purpose for which clinical information was originally collected did not strictly coincide with the objectives of the present study. An additional difficulty involved the absence of an informatic registry with the Basque Autonomic Justice Administration, as well as the lack of homogeneity of existing databases between public institutions, which would allow easier and rapid access to information of interest. To solve this, it was necessary to locate the files through the different courts in the Basque Autonomous Community and review them individually.

Despite the intrinsic technical challenges of this study, one of its main contributions has been the development of a multi-institutional collaboration for addressing the current, and possibly future, aims in understanding violence in first-episode psychosis, which had not taken place beforehand.

## Supporting information

Mentxaka et al. supplementary material 1 Mentxaka et al. supplementary material

Mentxaka et al. supplementary material 2 Mentxaka et al. supplementary material

## Data Availability

The data that support the findings of this study are available from the corresponding author, M.R.-B., on reasonable request.
