# Supplementary material for: Violence in first-episode psychosis: evidence from an early intervention in psychosis programme
Source: BJPsych Open. 2023 Sep 19;9(5):e172. doi: 10.1192/bjo.2023.564 (PMC10594161; doi:10.1192/bjo.2023.564)
Supplement: Mentxaka et al. supplementary material 1 — Mentxaka et al. supplementary material [file S2056472423005641sup001.docx]

**Appendix B - Supplementary material**

**Estimation of criminal rates**

For estimating crime rates, data on the number of known criminal offenses in relation to the total population were used. In our case, we calculated the annual crime rates for our healthcare catchment area (general population under care), as well as the annual crime rates for our cohort of FEP patients, in order to analyze whether these patients commit more criminal offenses compared to the general population.

The annual crime rates for the population in our healthcare service area were calculated by incorporating known criminal offenses reported by the security department of the Basque Country, taking into account the population from the official census provided by the National Institute of Statistics of Spain:

Criminality rate × 1000 inhabitants: total number of registered penal offences × (1000)

Population of study

For the specific case of the FEP patients, the annual crime rate was calculated using the total number of aggressions committed during each year and the sample of active patients in the CRUPEP intervention program in each year:

Criminality rate × 1000 FEP: total number of registered penal offences of FEP × (1000)

FEP cohort
