# Supplementary material for: Violence in first-episode psychosis: evidence from an early intervention in psychosis programme
Source: BJPsych Open. 2023 Sep 19;9(5):e172. doi: 10.1192/bjo.2023.564 (PMC10594161; doi:10.1192/bjo.2023.564)
Supplement: Mentxaka et al. supplementary material 2 — Mentxaka et al. supplementary material [file S2056472423005641sup002.docx]

**Supplementary material**

**Appendix A.** Main offences recorded as perpetrators or victims according to the Spanish Penal Code.

| **Violent Offences** | **Specific Criminal Code of the Spanish Criminal Code** |
| --- | --- |
| **Manslaughter and its forms**  Homicide | 138-141 articles |
| **Bodily harm**  Injuries  Intrafamily violence | 147-152 articles  153 article |
| **Robbery and burglary**  Burglary with force  Robbery with violence or intimidation  Theft or misappropriation of motor vehicles | 238 article  242 article  244 article |
| **On assaults on the Authority, its agents and civil servants, and on resistance and disobedience** | 550-556 articles |
| **On criminal offences against public health** | 359-378 articles |
